# An assessment of variation in quality of hypertension guidelines across income settings using the AGREE II tool

**DOI:** 10.12688/wellcomeopenres.22699.1

**Published:** 2024-09-17

**Authors:** Richu Philip, Carolina Janssen, Arun Jose, Thomas Beaney, Jonathan Clarke

**Affiliations:** 1 University Hospitals Birmingham NHS Foundation Trust, Birmingham, England, UK; 2 Imperial College London Department of Primary Care and Public Health, London, England, UK; 3 Amsterdam UMC Location AMC Department of Cardiology, Amsterdam, North Holland, The Netherlands; 4 Centre for Chronic Disease Control, New Delhi, India; 5 Imperial College London Department of Mathematics, London, England, UK

**Keywords:** Hypertension, Clinical Practice Guidelines, AGREE II, Guidelines, Evidence Based Medicine

## Abstract

**Background:**

Hypertension affects over one billion people worldwide, posing a significant global health burden. Clinical practice guidelines could play a key role in guiding healthcare providers in improving hypertension management. However, how the quality of hypertension CPGs differs across country income settings is not well understood. This study aims to explore variation in the quality of hypertension CPGs, comparing low-, middle-, and high-income countries, using the Appraisal of Guidelines for Research and Evaluation (AGREE) II tool.

**Methods:**

A Medline and grey literature search was conducted to identify hypertension CPGs in English from every country from January 2012 to September 2022. Two reviewers independently assessed and scored each CPG against the AGREE II tool. Results were described and the Kruskal-Wallis test was used to test for statistically significant difference in the domain scores across country income groups.

**Results:**

Forty-three CPGs were included for analysis from across income settings. Guidelines from HICs scored higher in four out of the six domains. The highest scoring domain was 4: “clarity and presentation” (median score 83%), the lowest scoring was domain 6 “editorial independence” (median score 0%). Statistically significant differences between income settings were observed for domain 3 “rigour of development” (p <0.001), domain 4 “clarity and presentation” (p = 0.03) and domain 6 “editorial independence” (p = 0.04).

**Conclusions:**

Whilst some variation exists in guideline quality across country income levels, the greatest degree of variation exists across the domains of the AGREE II tool. Global efforts to improve the quality of hypertension guidelines should focus on the transparent statement of editorial independence of guideline committees and apply rigorous replicable methods in the authoring of guidelines. Establishing national and international communities of practice to collaborate across income settings may reduce duplication of resource, allow for shared learning and promote the development of high-quality hypertension CPGs.

## Plain language summary

Hypertension is a medical condition that affects millions of people across the world and can lead to heart attacks and strokes. It is therefore very important that doctors can treat hypertension effectively. Clinical practice guidelines (CPGs) offer guidance to clinicians on how to treat hypertension. Our study aims to explore how the quality of hypertension CPGs varies, comparing low-, middle-, and high-income countries, using the Appraisal of Guidelines for Research and Evaluation (AGREE) II tool.

Our study compared the quality of forty-three CPGs from varying income levels. We found that guidelines generally scored well for their clarity and presentation, meaning they often provided specific, clear recommendations. Guidelines did not often clearly report who the authors work for and the sources of funding they’d received. Guidelines from high-income countries scored highest in four out of six domains of the AGREE II tool.

Whilst some variation exists in the quality of guidelines across country income levels, the greatest degree of variation exists across the domains of the AGREE II tool. Establishing national and international teams to collaborate across income settings may reduce duplication of work, allow for shared learning and improve the quality of hypertension CPGs.

## Introduction

As of 2019, hypertension affects over one billion people worldwide and accounts for over ten million premature deaths annually ^1^. The total number of people living with hypertension has doubled from 1990 to 2019, due to population growth and ageing, although the global prevalence of hypertension has stayed relatively consistent over the past three decades, with 34% of men and 32% of women between the ages of 30–79 years affected in 2019 compared to 32% of men and women in 1990 ^2^. However, this seemingly stable prevalence can be attributed to a decrease in prevalence in higher-income settings, with stagnant and in some cases starkly rising rates of hypertension in lower- and middle-income regions ^2^. Currently two-thirds of those affected by hypertension globally are from lower- and middle-income countries, posing a significant global health burden ^3^. The prevention and treatment of hypertension are key global targets for non-communicable diseases as set by the World Health Organisation ^3^.

Clinical practice guidelines (CPGs) are widely used by clinicians and could play an important role in the management of hypertension across the world ^4^. A CPG is defined as a document containing “systematically developed statements to assist practitioner decisions about appropriate health care for specific clinical circumstances” ^4^. Currently, there are several local, national and international CPGs for the management of hypertension that derive from differing income settings. The need for high-quality CPGs is important globally but considering the disproportionate burden of hypertension in low- and middle-income countries, may be even more pertinent in these settings.

Existing literature has found considerable variation in the recommendations made by hypertension CPGs between different income settings ^5^. Literature also suggests currently there is a higher quantity of hypertension CPGs from higher-income settings when compared to that from low- and middle-income settings. Currently there is limited research that compares the quality of hypertension CPGs from differing income settings ^6^. The few studies that do examine the quality of hypertension CPGs, are either entirely or predominantly focussed on high-income countries or include a very limited number of CPGs from low- and middle-income countries ^6, 7^. In earlier studies including CPGs from low- and middle –income countries, comparisons have been drawn between CPGs from these settings only, with no wider comparison made with CPGs from high-income countries ^8– 10^.

A well-recognised and widely used instrument that can be used to assess the quality of CPGs is the Appraisal of Guidelines for Research and Evaluation II (AGREE II) tool ^11^. The tool uses varying domains to assess guidelines developed by local, regional, national, or international groups or affiliated governmental organisations. Currently, it remains unknown whether there is variation in the quality of hypertension CPGs by income level, whether quality varies across different domains within guidelines or whether quality may vary across domains according to income level. Our study aims to explore variation in quality of hypertension CPGs across national and international settings, comparing low-, middle-, and high-income countries, using the AGREE II tool. This is an important avenue to explore, as it allows for a global perspective on the quality of existing hypertension CPGs across income settings. It can allow for identification of domains in which hypertension CPGs are performing well and identify areas which require improvement.

## Methods

### Search strategy

For this scoping review, a Medline search was conducted in September 2022 for articles that included the following combination of search terms in their title: “hypertension” OR “blood pressure” AND “guideline*”. In addition, a grey literature search was conducted using the Google search engine. This search was performed for every country recognised by the World Bank, including the search terms above plus the specific country searched for, e.g.: “hypertension” OR “blood pressure” AND “guideline*” AND “Angola”. The purpose of this search was not to be exhaustive but to allow for the inclusion of CPGs that may not have been published in an academic journal.

### Inclusion criteria

National and international CPGs published between January 2012 and September 2022, written in English, were included. Both CPGs specific to hypertension and general CPGs with guidance on multiple conditions were also included if they had recommendations for the diagnosis and management of hypertension. If a country had multiple guidelines, all were included provided they met the inclusion criteria. Where there were multiple versions of a CPG by the same governing body, the most recent version was included for analysis. Where partial updates of CPGs had been published, the previous full CPG was included.

### Data extraction

Two reviewers independently assessed each CPG against the AGREE II tool, the reviewers are a practicing clinician and an experienced health services researcher. An independent data extraction sheet was created using Microsoft Excel for each reviewer, including the domains and corresponding items of the AGREE II tool, in which the reviewer recorded their scores. Initially, both reviewers assessed the same six CPGs from varying income settings using the AGREE II tool and discussed their interpretation of scoring criteria. Subsequently, all included CPGs were assessed against the AGREE II tool and scores were determined for each of the 23 key items in 6 domains: ‘Scope and purpose’, ‘Stakeholder involvement’, ‘Rigour of development’, ‘Clarity of presentation’, ‘Applicability’, and ‘Editorial independence’. A seven-point Likert scale (1 being strongly disagree and 7 being strongly agree) was used to score each item. Items were scored independently by each reviewer and then compared; any item which had a score variation of two or more between the two reviewers were highlighted. The two reviewers met to discuss these differences to reach a consensus on the score and settled on one agreed score for the item. Any differences that were not resolved following this were discussed with a third reviewer to reach agreement on the score. 

Following this, the average percentage score for each domain for every CPG was calculated, according to the equation provided by the AGREE II tool, as follows:


$$\frac{\left(obtained\;score\;-\;minimum\;possible\;score\right)}{\left(maximum\;possible\;score\;-\;minimum\;possible\;score\right)}\;\times \;100$$

The AGREE II tool recommends that the user can determine how to interpret the results. For the purpose of this study, each domain was analysed separately. The AGREE II tool includes a final section labelled “overall guideline assessment”, on a seven point Likert scale where the reviewer can state whether they would recommend the guideline. This section was not included in this paper to avoid introducing bias on the part of the reviewers, as it was deemed subjective and provided no clear guidance on how to rate. This study classified countries based on country income data, such as specified by the 2022 World Bank classification of economies ^12^. The four categories are: low-income countries (LICs), upper-middle-income countries (UMICs), lower-middle-income countries (LMICs), and high-income countries (HICs). The Kruskal-Wallis test was used to compare whether there was a statistically significant difference in the domain scores across country income groups. Statistical analysis was conducted in Python (version 3.11, https://www.python.org) using the scipy package (version 1.12, https://scipy.org)

## Results

**Figure 1.**
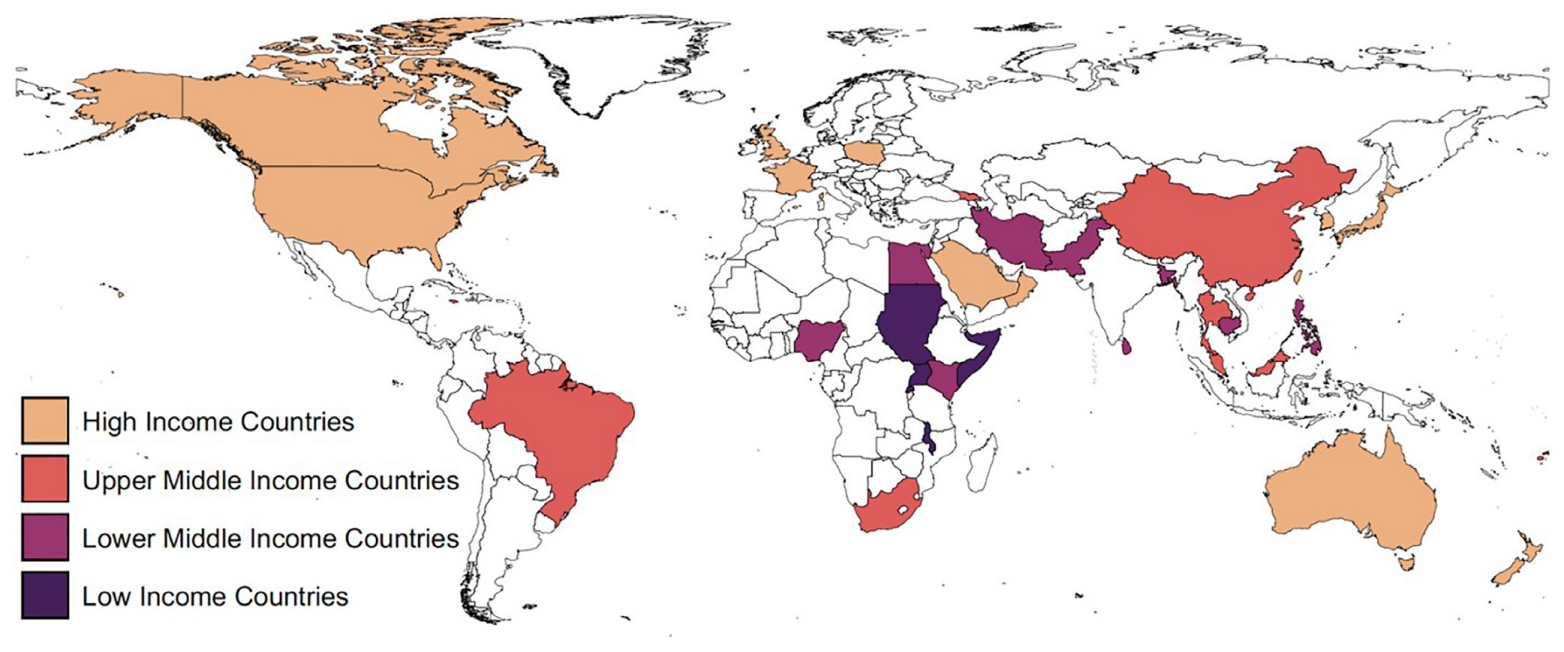
Map showing the geographic distribution of included countries, shaded according to World Bank income level. Shapefile source: https://gadm.org/index.html.

The Medline search result returned a total of 1182 results, of which 20 met the criteria for inclusion after review. Country specific grey literature search returned 23 CPGs that met the inclusion criteria. A total of 43 CPGs were included for analysis, of which 12% (n = 5) were from LICs (( 13– 17), 23% (n = 10) were from LMICs ^18– 27^, 19% (n = 8) were from UMICs (( 28– 35) and 40% (n = 17) were from HICs ^36– 52^. A further three guidelines, (6%) made recommendations that were applied to varied income settings ^53– 55^. The locations and income settings of included countries are shown in Figure 1.

### Overview of domains across all income settings

**Figure 2.**
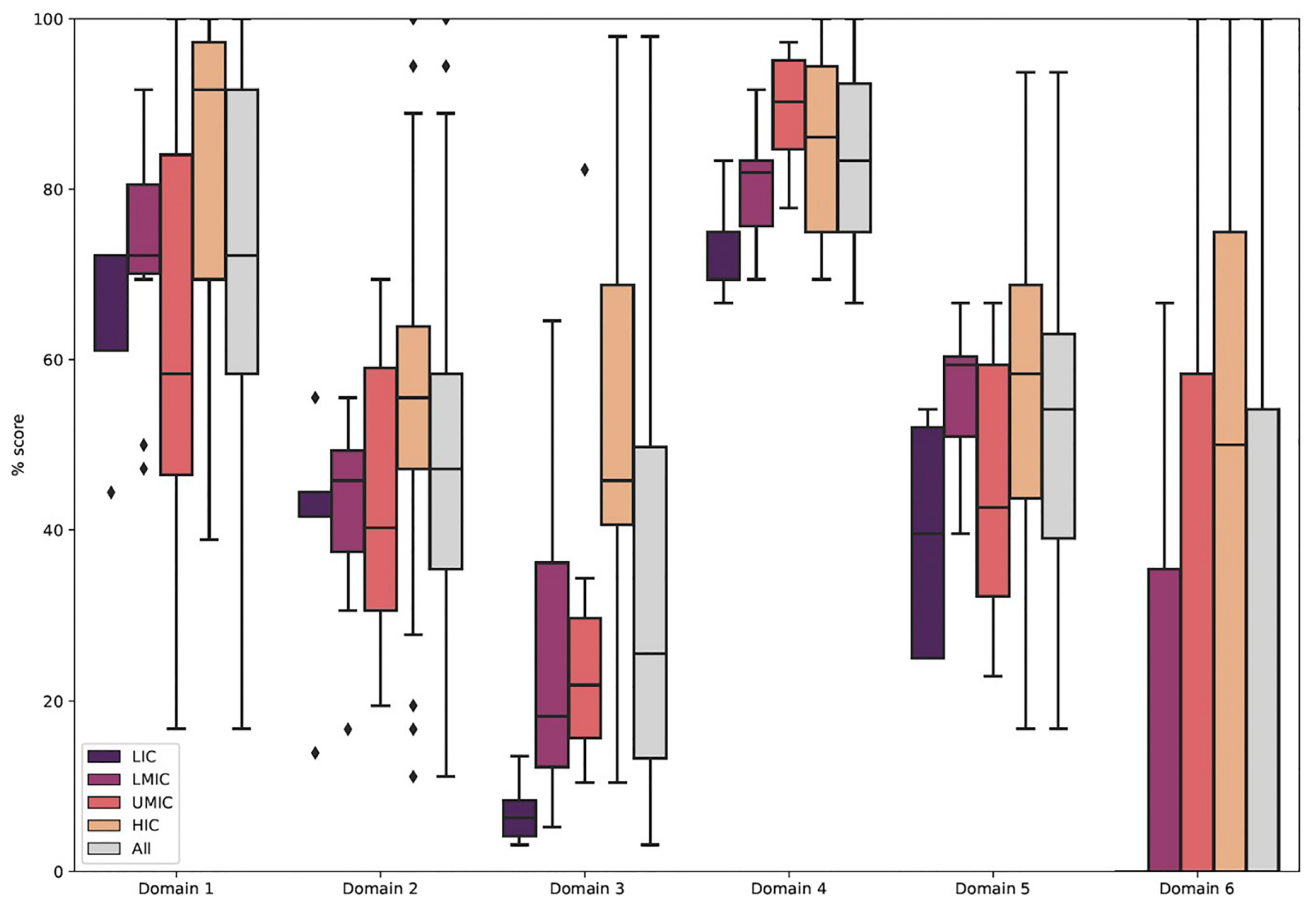
Boxplots showing the scores for each of the six domains of the AGREE II tool. Domain 1: “Scope and purpose”, Domain 2: “Stakeholder involvement”, Domain 3: “Rigour of development”, Domain 4: “Clarity of presentation”, Domain 5: “Applicability”, Domain 6: “Editorial independence”. LIC: Low-income countries, LMIC: “Lower-middle-income countries”, UMIC: “Upper-middle-income countries, HIC: “High-income countries.

Figure 2 shows the distribution of scores for each of the six domains overall and across income settings. Overall, the highest scoring domain was Domain 4: “clarity and presentation”, with a median score of 83%. The items in this domain focus on CPGs providing specific, unambiguous recommendations, easy identification of key recommendations and on the general presentation of the CPG. The next highest scoring was Domain 1: “scope and purpose” with a median score of 72% (IQR: 58–92%). This domain assessed whether CPGs provided clear objectives, outlined the health questions to be addressed and specified which patient population the CPG was relevant to.

The domain which scored lowest consistently across income settings was Domain 6 which related to CPGs declaring editorial independence, including each author’s affiliations and any funding sources. 53% of CPGs in this study (n = 23 / 43) scored 0% in this domain, as they did not provide an explicit statement detailing whether the CPG remained independent of external influence, nor declared the sources of their funding. The next lowest scoring domain was Domain 3, relating to the methodology and rigour of development of CPGs, which had the second lowest score with a median of 26% (IQR: 13–50%) across all income settings.

Of the remaining two domains, Domain 2 which scored CPGs for involvement of varied stakeholders including professional groups and patient representatives had a median score of 47% (IQR: 35–58%). Domain 5 of the AGREE II tool considers whether CPGs address barriers and facilitators, potential resource implications of recommendations, as well as practical tools on how the recommendations can be put into practice. The median percentage score for this domain was 54% (IQR: 39–63%).

### Income stratification

When stratifying the domains by income, statistically significant differences between income settings were observed for domain 3 “rigour of development” (p <0.001), domain 4 “clarity and presentation” (p = 0.03) and domain 6 “editorial independence” (p = 0.04).

The domain that showed the strongest evidence of difference in scores between income settings was with regard to the rigour and development of the guidelines, this addresses the methodology of CPGs (domain 3). In this domain, HICs had the highest median score at 46% (IQR: 41–69%), compared to only 6% (IQR: 4–8%) in LICs.

Of the remaining two domains that showed a statistically significant difference, in domain 4 “clarity and presentation”, UMICs had the highest median score with 90% (IQR: 85–95%), followed by HICs at 86% (75–94%), LMICs at 82% (76–83%) and LICs at 75% (69–75%). In domain 6 “editorial independence”, HICs had the highest median score at 50% (0–75%), UMICs, LMICs and LICs all had a median score of 0% for this domain.

Guidelines from HICs scored higher for scope and purpose (Domain 1) (median: 92%, IQR: 69–97%), than those from LMICs (median: 72%, IQR: 70–81%) and LICs (median: 72%, IQR: 61–72) or UMICs (median: 58%, IQR: 47–84%), although there was no statistically significant difference (p=0.18). HICs also scored the highest for domain 2 “stakeholder involvement” (median: 56%, IQR: 47–64%), this was also not statistically significant (p = 0.24).

No clear income gradient trends were seen for domain 5 “applicability”, with LMICs scoring the highest median at 59% (IQR: 51–60%), followed by HICs at 58% (IQR: 44–69%), UMICs at 43% (IQR: 32–59%) and LICs at 40% (IQR: 25–52%). No statistical significance was seen in this domain (p = 0.07).

## Discussion

This study identifies extensive variation in the quality of CPGs across different domains of the AGREE II tool, to a greater extent than variation between differing income settings of CPGs. CPGs tend to score similarly well in certain domains and score poorly in general across others, indicating that CPGs from all income levels have scope for improvement. Whilst CPGs from HICs generally tended to score better in each domain than those from lower income settings, there was no clear and consistent gradient observed with respect to income. Three domains showed a statistically significant difference in scores from CPGs across different income settings, with the strongest evidence for domain 3 “rigour of development” and some evidence for domain 4 “clarity and presentation” and domain 6 “editorial independence”.

CPGs generally scored well across all income settings in domain 4 “clarity and presentation”. This finding is concurrent with previous studies assessing CPGs against the AGREE II tool, which have also found this domain to be amongst the higher scoring ^7, 56^. Several existing studies assessing CPG use amongst clinicians have found usability as a highly important factor ^57, 58^, valuing a document that is easy to access and decipher. During data collection it was noted that several of the CPGs resembled academic journal articles rather than readily readable clinical documents. In general, CPGs from LICs and LMICs were shorter and quicker to read, which may make them more accessible for clinical use. Similarly, CPGs that offered summarised versions of their guidelines in addition to a detailed version were easier to gain information from quickly and more user friendly ^38, 40, 49^. Another domain which generally performed well across all income settings was domain 1 “scope and purpose”. Most CPGs included some definition of an introduction, thereby detailing its objectives and aims, meaning most CPGs scored well within this domain.

Overall, the worst performing domain across income settings was domain 6, relating to editorial independence. This is in line with other studies that report an overall lack of awareness of the need for reporting editorial independence across guidelines, where more than half of the evaluated guidelines failed to include a conflict of interest statement or declaration of financial support ^59, 60^. It may be the case that this is an aspect that guideline producers did not consider including or explicitly mentioning, as opposed to indicating a lack of editorial independence. Moreover, disclosure of interests alone is not sufficient in reducing the risks of bias in decision-making. Recent work has shown that members of guideline committees who have declared a conflict of interest, may paradoxically provide biased advice as they feel they have already informed the recipients of this risk ^61, 62^. Similarly, existing literature suggests that financial conflicts are associated with recommendations and outcomes that are biased toward the sponsor even where the conflict has been disclosed ^63^.

Within domain 3 “rigour of development”, CPGs from lower income settings were less likely to specify the methodology and process of developing the guideline and, in many cases, this was entirely omitted. These guidelines often had little explicit rationale or explanations behind their choice of recommendations, which led to lower scores in this domain when compared to HICs. Literature suggests that trust in the evidence-base that informs recommendations is an important influencing factor for clinicians when choosing a guideline. A qualitative study investigating clinician’s use of hypertension CPGs across different income settings found that most participants expressed greater trust in international guidelines, when compared to local or national guidelines, citing greater confidence in how they were formed, and placing high value on rigorous scientific trials from which recommendations were based ^58^.

Furthermore, several of the CPGs from lower income settings adopted recommendations from HIC CPGs to their particular setting. For instance, a CPG from Iran included an explicit and detailed methodology involving a search for a quality assessment of existing hypertension CPGs, using the AGREE II tool and subsequently selected NICE, JNC7 and Hypertension Canada guidelines on which to base their recommendations ^26^. A limitation to using the AGREE II tool when scoring CPGs that adopted this strategy is that they are penalised despite having evidence-based recommendations for not having undertaken their own evidence synthesis. It is known that the process of developing a CPG is expensive and time-consuming, and therefore it could be considered unrealistic to expect a comprehensive literature review and methodology for every guideline ^64^. However, whilst adaptation of guidelines may be a cost-effective strategy, there is concern regarding the representativeness of evidence arising from high-income settings to other income settings, particularly when considering implementation which may not be possible in lower income health systems ^65^. This applicability of CPGs is assessed within domain 5 of the AGREE II tool. Whilst no statistically significant difference was seen between income settings within this domain, there is scope across income settings for CPGs to better advise on practical implementation strategies, as well as address the resource settings, health care structure and the specific context in which their CPG is to be used. The International Society of Hypertension (ISH) guideline is an example of a CPG included in our study that acknowledges the tension between the latest evidence-based practices and the resource constrains of health systems, providing two sets of recommendations that may be applied depending on the local context.

When considering domain 2 “stakeholder involvement”, it is interesting to note that only 9% of CPGs scored better than ‘strongly disagree’ for the item specifying whether “the views and preferences of the target population (patients / public) have been sought”, making it one of the worst performing individual items within a domain. Evidence suggests that patient-orientated CPGs can provide valuable information surrounding patient perspectives, habits, preferences and values, thereby informing recommendations ^66^. However, in addition to being resource intensive, its incorporation also requires CPG developers with appropriate training and knowledge in qualitative research ^66^. Generally, this finding indicates a relatively insular process of guideline generation, where meaningful engagement of patients is the exception rather than the norm.

### Strengths and limitations

To our knowledge, this is one of few studies that have evaluated the quality of hypertension CPGs across varied national income settings. In comparison to existing studies within this field, this study includes a higher number of guidelines, particularly from lower income countries.

This study has several limitations. Firstly, this study only includes CPGs that were written in English. It may also include guidelines written in English from countries where English is not the language used in everyday clinical practice and for which guidelines in languages other than English may be more widely used. Secondly, the MEDLINE database search only included guidelines that specifically include the key terms “hypertension” and/or “blood pressure” and “guideline” in their title. Guidelines that failed to explicitly include these terms in their titles, were not published in a scientific journal, or did not identify as guidelines, might have therefore been missed. Whilst we tried to mitigate this limitation by conducting a grey literature search, we acknowledge that as the same search terms as above were used, we may have missed CPGs titled using different key terms e.g. “protocol” instead of “guideline”. We acknowledge that within the scope of our study, it was not feasible to exhaustively examine all eligible online CPGs for all countries. However, the aim of this study was not to identify all eligible guidelines, but to identify any variations in CPG quality between and across income settings.

All of the guidelines included are national or international in scope, and therefore we are unable to examine differences in the quality of guidelines used at local levels within nations. We expect this may be particularly important in health systems where intra-national differences exist in the capabilities of health systems. We also acknowledge that World Bank income levels are assigned to countries and therefore do not reflect the extent to which income inequalities exist within nations.

Thirdly, some CPGs include internal assessment of the guideline against the AGREE II tool as part of the authorship process. Though this might be expected to lead to high AGREE II scores which could bias assessment of quality, there were still significant areas for improvement even for guidelines where this tool was explicitly used during guideline development

### Recommendations for policy/practice

Our study identified weaknesses in the reporting of the methodology and process behind hypertension CPG development, which should be made clearer within guidelines. While creating new guidelines may be costly and time-consuming, particularly in lower income settings, clarification of the rationale behind recommendations would add clarity and transparency to users, for example, to allow an assessment of the degree to which guidelines are based on adaptations of international guidelines or tailored to the local context. However, this needs to be balanced against the usability of the guidelines, which could be hampered by including very detailed methodological text. Therefore, we recommend the use of summary documents for clinical use, with separate documents or access links detailing the methodology of guideline development, to aid usability and clarity. We also found that declaration of editorial independence was poorly recorded across guidelines, and is a crucial area for improvement for hypertension CPGs in the future.

Moving forward, it would be advantageous to establish national and international communities of practice to collaborate across income settings, to avoid duplication of resources and to share experiences in the development and implementation of CPGs. However, this must be executed carefully, and a clear distinction should be made between what can be generalised across income settings, and which should be adapted to local contexts ^67^. Such collaborations may be supported by recent, development of checklists for the adaptation of guidelines to different healthcare contexts, such as the RIGHT-Ad@pt Checklist ^68^.

## Conclusion

While we found some evidence of variation across country income levels in the quality of hypertension CPGs globally, the greatest degree of variation exists across the domains of the AGREE II tool. Our study demonstrates the global need for improvement in quality of CPGs, particularly across certain areas such as declaration of editorial independence and clearer detailing of methodology and process of guideline development. Considering the global burden posed by hypertension, particularly in low and middle-income settings, careful execution of national and international collaborative initiatives can aid in the sharing of experiences and ideas, utilisation of evidence-based research and avoidance of duplication of work when developing hypertension CPGs. This can aid in improving the quality of hypertension CPGs globally, as well as spearheading future work to understand the real-world utility and impact of these guidelines in clinical settings.

## Declarations

### Ethics and consent

No formal ethical approval was required for this study as it utilises evaluation of publicly available information.

### Consent for publication

Not applicable

## Data Availability

The datasets used and/or analysed during the study are published and available from the following repository: Zenodo: Additional material for study: Variation in quality of hypertension guidelines across income settings using the AGREE II tool- a scoping review. https://doi.org/10.5281/zenodo.12812653 ^69^. This project contains the following underlying data: Summary scores for each guideline Zenodo: PRISMA -ScR scoping review checklist for “Additional material for study: Variation in quality of hypertension guidelines across income settings using the AGREE II tool- a scoping review”. https://doi.org/10.5281/zenodo.12812653 ^69^. Data are available under the terms of the Creative Commons Attribution 4.0 International license (CC-BY 4.0) ( https://creativecommons.org/licenses/by/4.0/).
