# Supplementary material for: An assessment of variation in quality of hypertension guidelines across income settings using the AGREE II tool
Source: Wellcome Open Res. 2024 Sep 17;9:526. [Version 1] doi: 10.12688/wellcomeopenres.22699.1 (PMC11599801; doi:10.12688/wellcomeopenres.22699.1)
Supplement: Peer review history

# Reviewer report: Reviewer response for version 1

**DOI:** 10.21956/wellcomeopenres.25000.r101623

**Published:** 2024-11-26

**Authors:** Garry Jennings, Daniella Dougherty

**Affiliations:** 1 National Heart Foundation of Australia, Melbourne, Australia; 2 Clinical Evidence, Heart Foundation Adelaide, Adelaide, South Australia, Australia

This systematic reviewed looks at the quality of hypertension CPGs across different income settings using the AGREE II. The rationale was that there is a disproportionate burden of hypertension in LMIC but potentially lesser quality of guidelines. This question is topical and important given the large number of hypertension guidelines presently used around the world including two new European Guidelines released this year and forthcoming US AHA/ACC Guidelines in 2025.  The study included 43 CPGs identified through a Medline search and grey literature search of Google from 2012 to 2022. Guidelines from high-income countries generally scored higher, particularly in the domain of rigour of development. The study highlights significant variations in guideline quality across the global wealth divide, with the lowest scores observed in the domain of editorial independence.  This is not to say there was no editorial independence in all cases but not stating this aspect clearly in the guideline will always raise doubts.  It emphasises the need for global efforts to enhance guideline transparency and methodological rigour.

 The rationale and objectives of the systematic review are clearly stated. The researchers aim to explore the variation in the quality of hypertension CPGs across low-, middle-, and high-income countries using the AGREE II tool. The authors note that the global perspective is important because hypertension disproportionately affects low- and middle-income countries, and there is considerable variation in guideline recommendations by economic setting. By identifying the domains where CPGs perform well and areas needing improvement, the study provides valuable insights for designing and developing future guidelines in these regions.

 The authors have provided the search term combinations they used for the Medline search and the Google search terms for the grey literature, along with the date the search was conducted. The terms included ‘hypertension’ and ‘blood pressure’.  This could exclude guidelines that address primary or secondary prevention rather than single risk factors including hypertension in their title but may provide a similar level of guidance for clinicians on the management of hypertension to some specific hypertension guidelines.  The search was also confined to English language publications.

 The statistical analysis aligns with the nature of the data and the study's objectives.

 The study found statistically significant differences in guideline quality across income settings, particularly in the domains of "rigour of development," "clarity and presentation," and "editorial independence," with high-income countries generally scoring higher. The results showed that guidelines from lower-income countries often lacked explicit methodology and rationale, leading to lower scores in rigour. The authors acknowledge that the AGREE II tool may downgrade guidelines that adapt recommendations without conducting their own evidence synthesis, which could bias the results against lower-income settings where resource constraints make such guideline work challenging. Despite this, the study effectively highlights areas where all CPGs could improve and suggests strategies for low- and middle-income countries to enhance guideline quality. Therefore, the conclusions about the need for global improvement in CPG quality and the benefits of international collaboration are well-supported by the findings presented.

 While the study provides valuable insights into the quality of hypertension clinical practice guidelines across different income settings, there are areas that could be improved. Expanding the search strategy to include multiple databases beyond Medline may uncover additional relevant guidelines and make the study more comprehensive. Clearly documenting both inclusion and exclusion criteria is also important; without this information, others may find it challenging to replicate the study accurately or assess the completeness of the guideline selection.

 Furthermore, as the authors reported not using an exhaustive search, the report could  provide a clear rationale for this decision and discussing its potential impact on the comprehensiveness of the results. A more detailed explanation of how they ensured that relevant CPGs were still adequately captured despite not conducting an exhaustive search. This would provide transparency and help readers better understand any limitations of the search strategy.

 These do not compromise the scientific acceptability of the excellent article, as the methods, data, and conclusions remain robust and well-supported.

Are the rationale for, and objectives of, the Systematic Review clearly stated?

Yes

Is the statistical analysis and its interpretation appropriate?

Yes

If this is a Living Systematic Review, is the ‘living’ method appropriate and is the search schedule clearly defined and justified? (‘Living Systematic Review’ or a variation of this term should be included in the title.)

Not applicable

Are sufficient details of the methods and analysis provided to allow replication by others?

Yes

Are the conclusions drawn adequately supported by the results presented in the review?

Yes

Reviewer Expertise:

Cardiovascular and secondary research methodology

We confirm that we have read this submission and believe that we have an appropriate level of expertise to confirm that it is of an acceptable scientific standard.

# Reviewer report: Reviewer response for version 1

**DOI:** 10.21956/wellcomeopenres.25000.r105195

**Published:** 2024-11-11

**Authors:** Fadi Charchar

**Affiliations:** 1 Federation University Australia, Ballarat, Victoria, Australia

This study is a well-structured and insightful examination with significant implications for global health and clinical guideline policies. Given the substantial global burden of hypertension, the importance of high-quality clinical practice guidelines (CPGs) for managing the disease is paramount. A major limitation of the study, however, is its focus solely on English-language guidelines, which may affect the generalizability of its findings.

 The methodology is robust, featuring a comprehensive literature search, well-defined inclusion criteria, and a transparent process for data extraction and scoring using the AGREE II tool. Although the AGREE II tool is widely accepted, some criticisms of its framework should be addressed to enhance the study’s critical perspective. While the manuscript provides an overview of each domain, readers might benefit from a more detailed examination of Domain 6 (editorial independence), as this was the area with the most pronounced variation.

 The limitations section is comprehensive but could further discuss potential selection bias from excluding non-English guidelines and explore how this limitation affects the generalizability of the findings. Additionally, elaborating on the challenges of comparing CPGs that are adapted or adopted from international guidelines could provide valuable context.

 While the discussion of findings is well-developed, the authors could consider adding a section on the practical implications of guideline variation for healthcare providers which would be beneficial. Specifically, an exploration of how guideline quality impacts clinical decision-making in low- and middle-income settings—where resources and capacity are often limited—would add considerable value.

 In the discussion of Domain 2 (stakeholder involvement), consider expanding on the importance of including patient perspectives in CPG development. Incorporating patient input is essential for crafting guidelines that are both practical and ethically grounded, especially in settings where cultural or socioeconomic factors heavily influence healthcare practices.

 Minor revisions are recommended to improve sentence flow and reduce redundancy in sections such as the introduction and discussion.

Are the rationale for, and objectives of, the Systematic Review clearly stated?

Yes

Is the statistical analysis and its interpretation appropriate?

Yes

If this is a Living Systematic Review, is the ‘living’ method appropriate and is the search schedule clearly defined and justified? (‘Living Systematic Review’ or a variation of this term should be included in the title.)

Not applicable

Are sufficient details of the methods and analysis provided to allow replication by others?

Yes

Are the conclusions drawn adequately supported by the results presented in the review?

Yes

Reviewer Expertise:

Hypertension, Genomics, Lifestyle modifications

I confirm that I have read this submission and believe that I have an appropriate level of expertise to confirm that it is of an acceptable scientific standard.

# Reviewer report: Reviewer response for version 1

**DOI:** 10.21956/wellcomeopenres.25000.r105198

**Published:** 2024-10-22

**Authors:** Nicholas Jones

**Affiliations:** 1 Nuffield Department of Primary Care Health Sciences, Oxford, UK

Thank you for asking me to review this systematic review of the quality of international hypertension guidelines. 

**Major comments:**

 - I'm interested that you choose to limit the search to English language studies when this explicitly meant to be an international review - thank you for acknowledging this in the Limitations. Please consider explaining why you made this decision and also whether you think there might be implications about the type of guideline that you picked up (and missed) as a result? 

 - The search is limited to guidelines that use the term hypertension or blood pressure and I wonder if this might have risked missing other important guidelines e.g. the '2021 ESC guidelines on cardiovascular disease prevention in clinical practice' springs to mind. Again, thank you for acknowledging this as a limitation, but it does make me question what the purpose of the review is if not to be exhaustive? 

 - No mention is given re; a screening process of guidelines; please can you outline how this first stage of screening was done (reducing number of papers from 1182 to 20), including whether in duplicate or not and based on what assessment criteria? 

 - How did you handle continent-wide guidelines or international guidelines eg. ESC guidelines? This is probably the most widely used guideline in Europe and I see is included in the list of HICS guidelines. Figure 1 then feels a bit misleading to me as it seems most countries in Europe don't have a hypertension guideline but they are working from this shared document. 

 - It is over 2 years since the search was completed - this is a very long time ago and most reviews would aim to have a search update done within six months of publication. Some of the major guidelines you refer to have been updated - meaning the findings you present may already be out of date? 

 - You mention you included CPGs that were not only focused on hypertension management - it might be interesting to report whether there was any difference in reporting in these guidelines compared to hypertension specific CPGs. 

 - There has been some criticism of the AGREE II tool and a new AGREE-REX in JAMA Netw Open in 2020 (see  doi:10.1001/jamanetworkopen.2020.5535) - did you think about using this instead and could you justify your choice of tool? This may be of particular importance for your tool as AGREE II has been criticised for failing to capture the importance of context in implementation of guideline recommendations. 

 - There is a possible assumption in the review that people use the guideline from the country they live in, which I suspect is often not true. Established international guidelines tend to be referred to and worked against internationally. The purpose and intention of local guidelines may therefore be different. 

 - The review does not consider the healthcare setting where the guideline is meant to be applied - were any of the guidelines specific to primary or secondary care for example? 

 - I would be interested to know whether guideline quality has changed over time - are you able to comment upon this? 

**Minor** - please make clear CPGs refers to Clinical practice guidelines after first use in the abstract.

Are the rationale for, and objectives of, the Systematic Review clearly stated?

Yes

Is the statistical analysis and its interpretation appropriate?

Partly

If this is a Living Systematic Review, is the ‘living’ method appropriate and is the search schedule clearly defined and justified? (‘Living Systematic Review’ or a variation of this term should be included in the title.)

Not applicable

Are sufficient details of the methods and analysis provided to allow replication by others?

Partly

Are the conclusions drawn adequately supported by the results presented in the review?

Yes

Reviewer Expertise:

Primary care, cardiovascular disease, epidemiology.

I confirm that I have read this submission and believe that I have an appropriate level of expertise to confirm that it is of an acceptable scientific standard, however I have significant reservations, as outlined above.
